# Do biometric payment systems work during the COVID-19 pandemic? Insights from the Spanish users' viewpoint

**DOI:** 10.1186/s40854-021-00328-z

**Published:** 2022-03-09

**Authors:** Francisco Liébana-Cabanillas, Francisco Muñoz-Leiva, Sebastián Molinillo, Elena Higueras-Castillo

**Affiliations:** 1 Department of Marketing and Market Research, Faculty of Economics and Business Administration, University of Granada, 18011 Granada, Spain; 2 Faculty of Economics and Business, Department of Business Management, University of Málaga, Campus El Ejido, 29013 Málaga, Spain

**Keywords:** Biometric payment systems, Intention to recommend, COVID-19, Fear

## Abstract

Technological developments are changing how users pay for goods and services. In the context of the COVID-19 (coronavirus disease 2019) pandemic, new payment systems have been established to reduce contact between buyer and seller. In addition to the pandemic, the future is payment processing is also uncertain due to the new EU security regulations of the Payment Services Directive (PSD2). Biometric payments one option that would guarantee the security of transactions and reduce the risk of contagion. This research analyses the intention to recommend the use of the mobile phone as a tool for collecting payments in a shop using iris reading as a biometric measure of the buyer. The moderating effect of the fear of contagion in the proposed relationships was also analysed. An online survey was carried out, which yielded a sample of 368 respondents. The results indicate that the main antecedents of intention to use, which precedes intention to recommend, are perceived trust, habit, personal innovativeness and comfort of use. Additionally, the moderating effect of COVID-19 was checked among users with a higher perception of risk. The results obtained have interesting implications for purchase management among manufacturers and retailers.

## Introduction

COVID-19 (coronavirus disease 2019) is an infectious disease caused by the SARS-CoV-2 virus and was first officially detected in the Chinese city of Wuhan (Hubei province) in December 2019. Since its discovery up to July 2021, more than 190 million citizens have been infected worldwide, and more than 4 million have died (World Health Organization [WHO] 2021). Scientists have proposed numerous systems to prevent infection, including hygiene measures and maintaining social distance (Cheng et al. 2020; WHO 2021). This global situation has affected the use of cash and other alternative means of payment. While the European Central Bank or the WHO cannot prohibit or alarm citizens on this issue, past research has already warned of the possibility of disease transmission via banknotes and coins (Angelakis et al. 2014). More recent research by Kampf et al. (2020) and Van Doremalen et al. (2020) has shown the prevalence of coronaviruses on inanimate surfaces, suggesting that there is also a probability of transmission via banknotes and coins. Thus it is recommend banks focus on payment and money transferring alternatives (Kou et al. 2021). As well as, government institutions around the world have encouraged replacing cash with alternative payment methods to avoid the transmission of the disease as much as possible. As a result, the use of digital and contactless payments has accelerated (Panetta 2021)

The European Union’s new Payment Services Directive (PSD2)^1^ o regulating electronic payments requires companies and financial institutions to use at least one of the following three authentication systems: (1) something that only the user knows (password or security pin); (2) something that the customer has (validation by accepting a code communicated to the user’s mobile phone); and (3) something inherent to the user himself (iris or fingerprint). This new regulation will exclude payments made solely with card details.

In this context, new payment methods should emerge to make, as the PSD2 states, payments safer and more secure. One of the most innovative technologies, with the greatest growth potential, is based on the use of biometric techniques, with a forecast that by 2025 it will be used to authenticate more than $3 trillion payment transactions, up from just $404 billion in 2020—an increase of 650% (Juniper Research 2021). Kumar and Ryu (2009) define a biometric system as a pattern recognition system that collects biometric data from an individual, extracts a set of characteristics from the collected data and compares this set of characteristics with the template established in the database. Currently, biometrics technologies for banking and financial services such as face, finger, hand, iris and voice recognition are commercially available (Tellez and Zeadally 2017a; b). Companies such as American Express, Visa and BNP Paris are already developing payment systems with this technology, and customers are already using the facial recognition software Alipay to pay at Kentucky Fried Chicken (KFC) restaurants in China and Russia (Findbiometrics 2021). Biometric payment systems have many advantages, including high service speed and security, as well as convenience, because consumers do not have to carry cards or mobile devices, unlike other online payment systems (Zirjawi et al. 2015). However, the level of adoption of these technologies is still very low, even in developed areas such as North America and Europe (Stickland 2018). As a result, methods such as automatic cluster detection are being applied to infer user behaviours in various financial applications (Li et al. 2021).

Knowing the factors that influence consumer intention to use these technologies would help companies to design the devices in a way that would favour their adoption. Despite its potential usefulness, academic research has focused mainly on technical aspects for developing the technology, while studies on the adoption of and intention to use for biometric payments systems remain very limited (Kim et al. 2019; Li et al. 2020). Some researchers have analysed the consumer perspective on fingerprint recognition technologies (e.g. Ogbanufe and Kim 2018) and facial recognition (e.g. Liu et al. 2021). According to the literature, the factors that influence the intention to use these biometrics payment systems are related to the perception and attitude towards the pros and cons of the technology. These factors often include perceived usefulness, ease of use, risk, trust, convenience, social influence or personal innovativeness (see Kim et al. 2018; Liu and Tu 2021; Moriuchi 2021). However, to the authors’ knowledge, the antecedent factors of the intention to use payment systems based on iris recognition have not yet been studied. This technology provides the highest security in user authentication (Modibbo and Aliyu 2019) and, unlike other technologies such as fingerprints, requires no physical contact with a device for use. However, its adoption is not without drawbacks, as potential users are often wary due to the fear that infrared rays may adversely affect vision (RecFaces 2020). Therefore, explanatory factors for intention can be expected to differ from those of other biometric payment methods studied so far. The present study will address the following research questions:

- RQ1: What are the key factors for consumers’ acceptance of biometric systems, in particular mobile payment with iris scans, for purchases?
- RQ2: Has the COVID-19 pandemic affected the intention to use and recommend these biometric mobile payment systems?

To answer these research questions, we adopted an environmental psychology perspective (Mehrabian and Russell 1974) and used the stimulus-organism-response (S-O-R) model. The S-O-R paradigm has been used to analyse consumer behaviour in the face of new technologies such as mobile payments (Chen et al. 2019), mobile commerce apps (Chopdar and Balakrishnan 2020), social commerce (Molinilo et al. 2021) and virtual reality in tourism (Kim et al. 2020), among others. It would allow us to analyse the background of the intention to use iris recognition payment systems and to build an integrated model that would reflect how the use of this technology could lead to an increase in this type of payment.

The purpose of this research is fourfold. First, it addresses a gap in the current literature on biometric payments and more specifically on iris scan systems. Second, the study proposes the application of the stimulus-organism-response (S-O-R) framework to analyse the importance of various factors in explaining the intention to use payment systems based on iris recognition technology. Third, we explore the effect that the COVID-19 pandemic has had on biometric payment systems when cash is eliminated, as well as the physical contact between buyer and seller with the use of a point of sale (POS) transaction. Finally, policymakers can use the results to strengthen their understanding of payment innovation and to improve policies relating to adopting this technology and its positive effects on society. The present study thus makes an original and novel contribution to the theory and practice of means of payment by improving knowledge about the factors that influence the intention to use and recommend an emerging technology through the evaluation of a behavioural model that includes relationships and a hitherto unexplored moderating effect.

This study continues as follows: section two defines the key terms used in the research, summarises the relevant previous research and outlines the theoretical framework. The proposed research model and research hypotheses are presented in section three. Section four describes the methodology used for data collection, questionnaire design and analysis. Sections five and six provide the results of the analysis and discussion together with the implications, limitations and future lines of research, respectively.

## Evolution of biometrics: iris recognition payment systems

Recent technological developments have meant that traditional payment systems (cash or bank cards) are starting to lose importance and are being replaced by new contactless payment systems using new media (contactless cards or mobile phones). Biometric payments have therefore become an opportunity for both users and merchants. Biometric technology can be defined as “a way of personal identification using the psychological or the behavioural characteristics” (Awad 2012: 524). According to Burt (2018), the Goode Intelligence report predicts that “there will be 2.6 billion people using biometric payments by 2023, driven by demand for frictionless authentication in all channels, the need to reduce payment fraud, regulation at both the state and industry level, and technology standardization.”

To overcome the limitations of conventional methods, advanced authentication schemes have been developed for mobile computing systems to provide usable security to consumers (Jain et al. 2000; Crawford et al. 2013). In particular, biometric technology based on personal uniqueness (i.e., physical traits) is being successfully used to provide secure and usable user authentication (Clodfelter 2010; Choi et al. 2020). Despite the large number of biometric systems that can identify users, the main payment systems using biometrics as a medium have incorporated fingerprint recognition, finger vein recognition, facial recognition and iris recognition scanning (Hartoneva 2020). These payment systems have mostly been studied from a technical perspective, but without delving into behavioural aspects (Jiang and Li 2020; Tymoszek et al. 2020). Table 1 summarises the different characteristics of biometric technology proposed by Liu and Silverman (2001) in Modibbo and Aliyu (2019).

**Table 1:** Comparison of biometric technologies

Characteristics	Fingerprints	Hand-geometry	Retina	Iris	Face	Signature	Voice
Ease of use	High	High	Low	Medium	Medium	High	High
Error incidence	Hand injury, age	Hand injury, age	Glasses	Poor lighting	Lighting, age, glasses, hair	Changing signature	Noise, illness, weather
Accuracy	High	High	Very high	Very high	High	High	High
Cost	*	*	*	*	*	*	*
User acceptance	Medium	Medium	Medium	Medium	Medium	Very high	High
Required security level	High	Medium	High	Very high	Medium	Medium	Medium
Long-term stability	High	Medium	High	High	Medium	Medium	Medium

The benefits of this technology are associated with security and the reliable identification of users (Yang et al. 2013; Misra et al. 2020). The proposed research analyses the most secure payment system according to the review carried out: scanning the user’s iris. This system allows payments to be made by scanning the user’s iris with a simple reader installed on the shop’s mobile phone. So far, no academic research has been carried out on the background and consequences of this type of payment, so analysis would be relevant. However, on a practical level, this type of initiative has been developed in environments less prone to the use of state-of-the-art technological solutions, such as the Syrian refugee camp of Azraq in Jordan, where it was possible for people to pay for purchases using their eyes on a technology based on Blockchain to guarantee the identity of each person and to subtract their purchases from the money allocated to them (El Confidencial 2020).

## The case of Spain

Regarding the use of biometrics in payment systems in Spain, the main payment system providers, such as MasterCard, are already developing specific applications to incorporate these techniques in payment scenarios, such as the use of voice recognition at ATMs or even in vending machines, which could allow customers to validate payment with their fingerprint without keys or passwords, or even using facial recognition in certain buying and selling relationships. According to Statista (2020), the biometric authentication technology with the greatest potential for acceptance among digital payment users in Europe between 2018 and 2022 is fingerprint payment (31%), followed by facial recognition (11%), iris scanning (10%), voice recognition (5%), palm vein recognition (4%) and others (4%). Within the new pandemic context, it is important to highlight payment systems in which no physical contact is made between customer and vendor and which allow the use of facemasks.

Along the same lines, the report “Mobile Payment Authentication & Data Security: Encryption, Tokenisation, Biometrics 2019–2024”, produced by Juniper Research (2019), states that biometric authentication will be used to secure mobile payments worth $2.5 trillion in 2024, compared to $228 million in 2020. If this forecast is met, there would be cumulative growth of 1000% in the period 2019–2024. Indeed, according to data from the Payment Innovation Hub^2^ (2020), more than 60% of Spaniards are willing to pay with biometric tools, which they perceive to be a safe, convenient and fast method.

## Theoretical framework development

The literature has proposed numerous behavioural models to explain the antecedents of intention to use as well as the use and recommendation of various means of payment. Most authors have followed the approaches of classical models of consumer behaviour such as the theory of reasoned action (Fishbein and Ajzen 1975), the theory of planned behaviour (Ajzen 1991), the technology acceptance model (Davis 1989), and the unified theory of acceptance and use of technology (Venkatesh et al. 2003). Although these studies have made important contributions to improving our knowledge of the variables that influence consumer behaviour, we consider that the use of different theoretical frameworks such as the S-O-R model could enrich both theory and practice in this field of study in which, so far, few authors have used it as a reference in their research (Yuan et al. 2020).

The S-O-R model proposed by Mehrabian and Russell (1974) establishes a series of environmental aspects that act as stimuli (S) which influence the internal affective and cognitive states of each person (O), leading them to conscious or unconscious behaviour (R). The use of the S-O-R model as a theoretical framework is appropriate for this study for two reasons. First, because the three-phase structured process (S-O-R) to explain consumer behaviour fits the decision-making process for using payment methods, as users are exposed to environmental stimuli (e.g. subjective norms, effort expectancy) that they process and evaluate internally before making the decision to use one payment method or another. For example, Kalinic et al. (2019b) have shown that the opinion of the social environment on peer-to-peer mobile payment systems influences the decision of potential users to use one means of payment or another. Second, recent studies have shown that the S-O-R model is suitable for addressing consumer behaviour in payment systems (Kim and Rha 2016; Chen et al. 2019; Yuan et al. 2020). For example, Chen et al. (2019) found that utilitarian value, hedonic value and salesperson behaviour towards mobile payments (S) influence user satisfaction (O), which in turn determines the intention to use mobile payments (R). Yuan et al. (2020) showed that the information quality, system quality and service quality of mobile payment systems positively influence satisfaction, trust and intimacy (O) and satisfaction and intimacy predict loyalty to the mobile payment system (R).

The present study uses the S-O-R model to provide a parsimonious and structured way to explore the effects of convenience, effort expectancy, subjective norms, personal innovation and habit as stimuli (S), and perceived usefulness and perceived trust as the organism state (O) for the intention to use and recommend iris recognition payment systems (R). In doing so, the proposed model is consistent with the literature on means of payment and makes several original contributions, both for evaluating the impact of variables so far little studied in this context (e.g. convenience and habit) and for the payment system itself (i.e. iris recognition) whose intention to use is analysed.

### Stimulus (S): convenience, effort expectancy, subjective norms, personal innovation and habit

As discussed above, in this study, the term environmental stimuli refers to stimuli factors for using biometric payment systems and can be considered an umbrella term for a range of factors in using iris recognition technologies. In this context, the present study evaluates the influence of five factors reflecting the external (technical and social) and internal environment of the individual. First, the convenience of using a payment method (e.g. that it is accessible anytime, anywhere) would seem an obvious prerequisite for users to have the intention to use a payment method (Teo et al. 2015a, b). Similarly, the effort the user believes he/she would have to make to use a payment method or how easy it seems to him/her will invite him/her to use it to a greater or lesser extent (Kim et al. 2010). Along with these two factors that are closely related to the technical characteristics of the payment method, the environment also includes the influence of a social factor that reflects how people close to the users affect the decision to use it (Kalinic et al. 2019b). In the internal environment of the individual, the greater or lesser predisposition of the individual to use emerging and unknown technologies will be fundamental, which in the payment media literature is known as personal innovativeness (Liébana-Cabanillas et al. 2021a, b). Finally, it is suggested that the individual’s habit of using new technologies will predispose him or her more favourably to the use of a new technology (Venkatesh et al. 2012).

The perceived convenience of an application refers to the extent to which consumers consider it to be desirable for the efficient performance of a task (Chang et al. 2013). The need for customer convenience is continuously increasing for many reasons, most notably those related to socio-economic change, the competitive market, technological development and rising opportunity costs. This variable and its relationship with improving intention to use have been empirically contrasted in different technology-related research (Loiacono et al. 2013; Mushwana and Maziriri 2017; Maziriri et al. 2020). It is in this sense that we suggest that users use the proposed payment system because it is more efficient than the use of other payment systems—that is, more convenient—which leads to the following research hypothesis:

#### H1:

Perceived convenience will have a significant positive impact on intention to use payment systems with ocular iris scanning.

According to the unified theory of acceptance and use of technology, effort expectancy is defined as the degree of ease associated with the use of the system (Venkatesh et al. 2003). Based on existing studies on the adoption of mobile payments, the expectation of effort is one of the most significant factors influencing the adoption of payment systems (Alalwan et al. 2017; Al-Saedi et al. 2020). Consequently, as Teo et al. (2015a) state, because mobile payment requires less physical and mental operational effort than traditional payment methods, the degree of perceived ease associated with the use of mobile payment is likely to affect the intention to use it; this leads to the following research hypothesis:

#### H2:

Effort expectancy will have a significant positive impact on intention to use payment systems with ocular iris scanning.

Subjective norms are defined as the degree to which individuals perceive that people who are important to them, think they should or should not use a system or take some action (Fishbein and Ajzen 1975). The influence of these people’s opinions will largely determine user behaviour (Lara-Rubio et al. 2020). In the opinion of many authors, a direct and positive link has been identified between subjective norms and perceived utility (Zhanga et al. 2011; Liébana-Cabanillas and Alonso-Dos-Santos 2017; Yasin et al. 2019). A direct relationship between subjective norms and trust has also been proposed, as trust is understood as a concept built socially—that is, based on the opinions of the people who are part of the user’s environment (Suntornpithug and Khamalah 2010; Kalinić et al. 2019a). Therefore, the following hypotheses are proposed:

#### H3:

Subjective norms will have a significant positive impact on perceived usefulness.

#### H4:

Subjective norms will have a significant positive impact on perceived trust.

In general, users’ capacity for innovation describes the extent to which individuals are attracted to new products or innovations and want to try and adopt them (Rogers 1983). The greater an individual’s capacity for innovation, the more favourable their intention to use a technology (Herrero-Crespo and Rodriguez del Bosque 2008; Kasilingam 2020). Indeed, other researchers have found that innovation is an important factor affecting the use of mobile payments (Thakur and Srivastava 2014; Kang 2019; Patil et al. 2020). In short, personal innovation is a key variable in the relationship with intention to use an ocular iris scanning payment system; hence, the following hypothesis was proposed:

#### H5:

Personal innovation will have a significant positive impact on intention to use payment systems with the ocular iris scanning.

De Guinea and Markus (2009) examined how habit is a key factor influencing the choice to continue using technology beyond rational decision factors related to the technology’s utility or characteristics. Habit is a behavioural pattern in which people engage because of their past learning (Verkijika 2018). Repetitive use will thus improve future use (Venkatesh et al. 2012; Jia et al. 2020). Users who have used some novel payment service (e.g. mobile payment) would experience the trend and convenience of using these applications to carry out their transactions (Pal et al. 2020), and the habit of using these services would favour the intention to use the proposed new payment systems. The following research hypothesis is therefore proposed:

#### H6:

Habit will have a significant positive impact on intention to use payment systems with ocular iris scanning.

### Organism (O): perceived usefulness and perceived trust

According to the S-O-R framework, the impact of stimuli on user behaviours could be mediated by the organism’s cognitive and affective processes (Mehrabian and Russell 1974). The literature on the adoption and use of payment methods has so far emphasized the importance of the user’s cognitive evaluation in explaining his or her intention to use a payment method (e.g. Kim et al. 2019; Liébana et al. 2021; Moriuchi 2021; Ramos-de-Luna et al. 2019). In contrast, some researchers have shown that affective evaluation in the short term, such as perceived enjoyment, is often less influential (Rouibah et al. 2016) or not significant (Koenig-Lewis et al. 2015), while in the long term it requires a cumulative process of experiences with the technology (Yuan et al. 2020) that cannot yet be measured for iris recognition payment systems. In this study we therefore focus on the individual’s cognitive evaluation. Among the various factors that could be considered, previous literature reviews on payment systems have highlighted the important roles of perceived usefulness and perceived trust for explaining the intention to use new payment systems (Alkhowaiter 2020; Karsen et al. 2019). The present study thus includes these two key factors in the research model as organism variables.

Perceived usefulness is defined as the potential user’s subjective sense of the probability that using a specific system will improve work performance in an organisational context (Davis et al. 1989). In the context of the adoption of mobile phone payment services, perceived usefulness refers to the extent to which people believe that a service will help them perform payment transactions conveniently (Phonthanukitithaworn et al. 2016). If a user perceives that a technology will help him to make payments more easily and quickly, he or she will believe that its use brings benefits in terms of saving time and effort and will consequently be more motivated to use it. This study therefore suggests that the perceived usefulness of the proposed payment system will enhance the intention to use it in line with previous research (Kalinić et al. 2019b; Liébana-Cabanillas et al. 2020a, b). The following research hypothesis is therefore proposed:

#### H7:

Perceived usefulness will have a significant positive impact on intention to use payment systems with ocular iris scanning.

In recent decades, research in the field of marketing has highlighted the importance of trust between parties as an instrument to promote the continuity of a relationship. This is an aspect of great importance in the business world (Dwyer et al. 1987). Although it is true that trust in virtual environments is a difficult concept to explain due to its complexity, some researchers attempt to do so through credibility or security (Wang and Emurian 2005). Perceived trust and risk are the factors most often addressed when assessing the acceptance of mobile payments, just behind ease of use and usefulness already proposed in this research (Dahlberg et al. 2015). Perceived trust relates to the security of mobile payments, confidentiality of personal data, reliability of the results of mobile payment transactions and integrity of the use of mobile services (Zarmpou et al. 2012). This study therefore assumes that improved confidence would influence consumer intention towards the adoption of these new payment systems (Kalinić et al. 2019b). The following research hypothesis is thus proposed:

#### H8:

Perceived trust will have a significant positive impact on intention to use payment systems with ocular iris scanning.

### Response (R): intention to use and intention to recommend

The individual’s response to environmental factors and cognitive exercises is his or her choice or decision that he or she manifests via personal behaviour (Mehrabian and Russell 1974). Research on consumer behaviour in relation to technology is usually aimed primarily at explaining and predicting their intention to use (Karsen et al. 2019; Verma et al. 2020) as this variable is considered a good predictor of actual technology use behaviour (Venkatesh et al. 2012). Along with intention to use, we also considered it appropriate to explore the intention to recommend the technology. Although technology recommendations have not been widely studied because more attention has been paid to the construct of usage behaviour, we consider such recommendations to be fundamental in the study of the proposed payment system. A recommendation is here seen as a post-adoption behaviour, making the intention to recommend a key factor for successful dissemination (Naranjo-Zolotov et al. 2019). Several studies provide evidence that users with a high level of intention to use a technology are more likely to recommend the given technology to other users (Miltgen et al. 2013; Oliveira et al. 2014, 2016; Talukder et al. 2019). We therefore believe that users who express an intention to use the proposed payment system will be more likely to recommend it to other users:

#### H9:

Intention to use will have a significant positive impact on intention to recommend payment systems with ocular iris scanning.

### Moderating effect of COVID-19

In addition to the above hypotheses, the study of the moderating effect of COVID-19 in the current pandemic situation is proposed as a highly topical issue. The social and economic consequences generated by the new economic, social and health conditions brought about by the COVID-19 pandemic has generated a feeling of fear among the world’s population. According to Nicomedes and Avila (2020) fear scores the highest in the scale of the Health Anxiety Inventory (HAI), and Mertens et al. (2020) defined fear as an emotion of adaptation that mobilises an individual’s energy to face a possible threat. Fear of COVID-19 can manifest in various ways; some people have stopped going out on the streets for fear of contagion, others use gloves to protect themselves against the virus and many others have stopped using cash entirely to avoid contagion. This new situation causes mental health problems, including stress and anxiety (Arora et al. 2020; Sauer et al. 2020) and behavioural changes (Al-Maroof et al. 2020). In this new context, many buyers have changed their habits to protect themselves based on two important issues: the high degree of concern and the high possibility of being affected by the disease (Ahorsu et al. 2020). For these reasons, we consider it essential to know the moderating effect of fear of COVID-19:

#### H10:

The fear of COVID-19 influences the proposed research model.

**Fig. 1:**
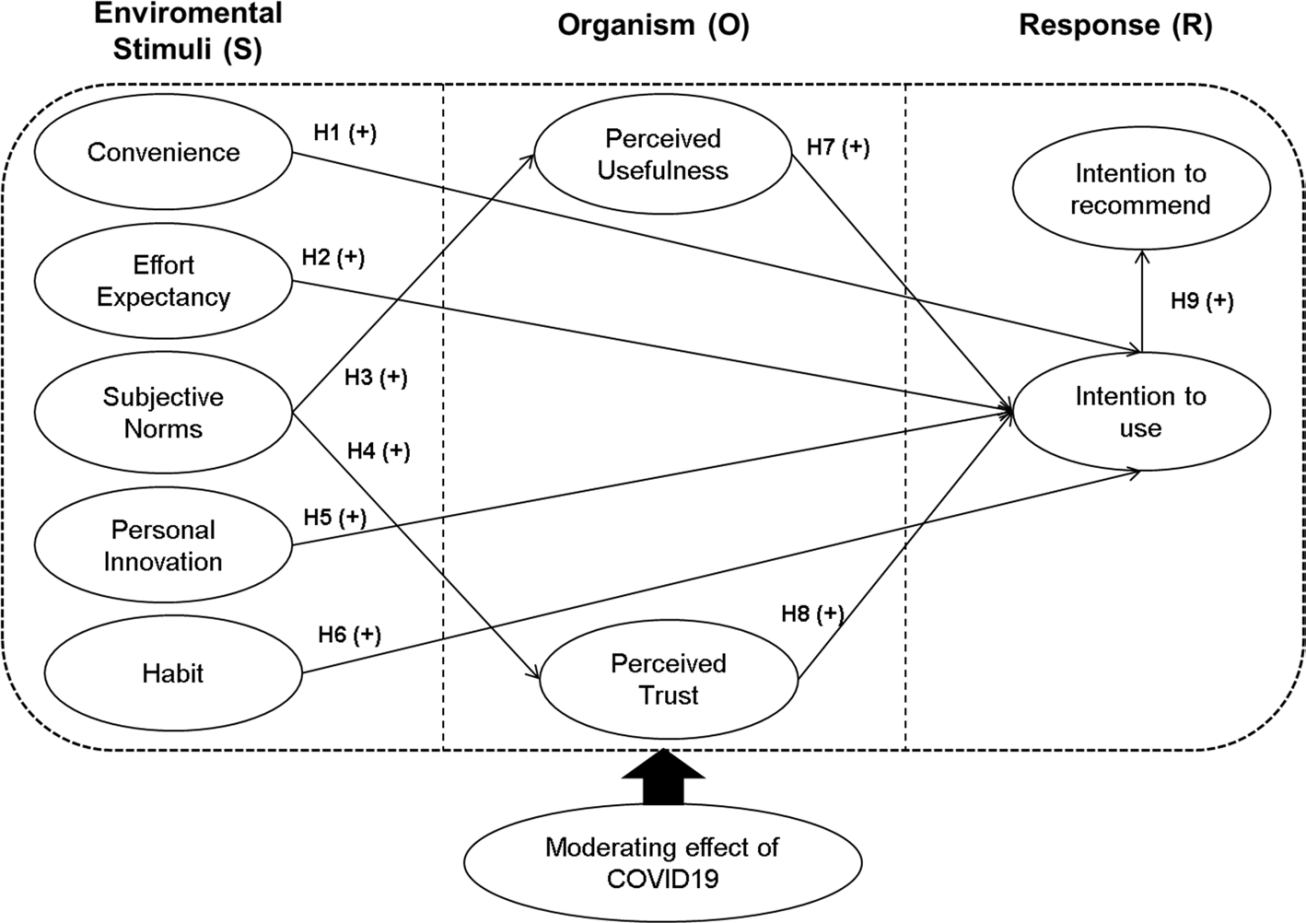
Research model

Based on the theoretical background, we present the research model shown in Fig. 1 to analyse the influential factors between variables.

## Method

### Data collection and measurement scales

An online survey was conducted in Spain to evaluate the proposed model. The survey was based on an adaptation of the following scales previously validated in the scientific literature: convenience (Pal et al. 2015; Liébana-Cabanillas et al. 2019); effort expectancy (Venkatesh et al. 2012; Hew et al. 2015); subjective norms (Taylor and Todd 1995; Kalinić et al. 2019); personal innovation (Ramos de Luna et al. 2016; Kalinić et al. 2019); habit (Cheng et al. 2020); perceived usefulness (Ramos de Luna et al. 2016); perceived trust (Zarmpou et al. 2012); intention to use (Liébana-Cabanillas et al. 2020b); and finally intention to recommend (Ma et al. 2017). All variables were measured on a 7-point Likert scale, where 1 represented “strongly disagree” and 7 “strongly agree”. As the original scales were in English, they were translated into Spanish by a native translator to ensure the accuracy of the content. Subsequently, a back translation was applied to ensure the quality of the translation (see “Appendix”). Fear was measured through a direct question to the respondent.

The final survey was conducted using Qualtrics, a PHP-based survey program used to create online surveys. To reduce the dropout rate, information on the purpose of the research was inserted and the anonymity of the individuals was guaranteed, as were data protection and non-use of the data for other purposes. A pre-test was conducted with 10 experts and 100 participants to ensure understanding of the questionnaire and its appropriateness to the objectives.

The participation process consisted of four stages. In the first stage, potential respondents were invited to participate in the research with a link to the questionnaire. Then, once users had accepted the invitation, they were provided with a link to a video explaining the concept of biometric payments and, more specifically, payments using ocular iris scanning. The video was designed to facilitate understanding of this novel payment system. To motivate the participants to watch the entire video, they were informed before watching it that they would have to provide a code after watching that would be shown at the end of the video. This research model has already been validated in recent research (Liébana-Cabanillas et al. 2018) and facilitates information being processed consciously or unconsciously to encourage recall, which improves the probability that participants will remember the messages shown and therefore increases the reliability of the results. In the third stage, after having watched the video, the participants were asked about their experience with current payment technologies (NFC, QR or P2P) and were asked to respond to the questionnaire. They were also asked to send their contacts an email inviting them to participate in the research.

The survey was carried out in September and October 2020, with an initial sample of 394, following a convenience sampling method. After a filtering process in which users who had responded with a pattern, incomplete responses, high or low response times and outliers were removed, the final valid sample was 368 users (93.40%); of these, 56.52% were women; 52% were aged between 18 and 24, 22.5% between 25 and 44 and 25.5% were over 44. Of the total, 49.93% had completed university studies, 26.68% had completed secondary education, 15.53% had completed primary education and the rest had completed no education (Table 2).

**Table 2:** Sample characteristics

	Number	%
**Gender**		
Male	160	43.48
Female	208	56.52
**Age**		
18–24	191	52.00
25–44	82	22.50
Over 44	95	25.50
**Education**		
No education	29	7.86
Primary education	57	15.53
Secondary education	98	26.68
University studies	184	49.93
**User**		
Yes	344	93.40
No	24	6.60

### Normality and common method bias

Normality tests were conducted based on the skewness and kurtosis values of each item (Table 3). These were below the absolute values of 2 and 7, respectively, which allowed us to use maximum likelihood procedures, which indicated similarity with the normal curve (Curran et al. 1996).

**Table 3:** Descriptive statistics, convergent validity and internal consistency reliability

Variables	Mean	VIF	Loadings	Kurtosis	Skewness	CA	Rho_A	CR	AVE
**Convenience**									
CON1	4.57	2.866	0.899	− 1.131	− 0.434	0.911	0.917	0.938	0.790
CON2	4.52	2.779	0.886	− 0.928	− 0.499				
CON3	4.73	3.250	0.908	− 0.928	− 0.410				
CON4	4.55	2.510	0.862	− 0.793	− 0.541				
**Effort expectancy**									
EE1	5.15	3.971	0.968	− 0.678	− 0.751	0.928	0.931	0.965	0.932
EE2	5.16	3.971	0.963	− 0.703	− 0.692				
**Subjective norms**									
SN1	3.98	2.334	0.867	− 1.108	− 0.164	0.891	0.893	0.925	0.754
SN2	3.63	2.809	0.892	− 0.999	0.161				
SN3	3.65	2.337	0.854	− 1.156	0.157				
SN4	3.98	2.315	0.861	− 1.163	− 0.079				
**Personal innovation**									
PIIT1	4.58	3.605	0.915	− 0.974	− 0.399	0.939	0.940	0.956	0.845
PIIT2	4.18	2.928	0.886	− 1.135	− 0.202				
PIIT3	4.56	4.719	0.938	− 0.891	− 0.356				
PIIT4	4.39	4.587	0.936	− 1.034	− 0.202				
**Habit**									
HAB1	4.15	3.109	0.925	− 1.103	− 0.139	0.901	0.916	0.938	0.834
HAB2	4.26	3.836	0.942	− 0.938	− 0.178				
HAB3	4.43	2.397	0.872	− 0.831	− 0.308				
**Perceived usefulness**									
PU1	4.45	1.960	0.819	− 1.026	− 0.301	0.889	0.899	0.923	0.751
PU2	4.55	2.802	0.891	− 0.884	− 0.357				
PU3	4.26	2.348	0.861	− 1.010	− 0.163				
PU4	4.58	2.591	0.893	− 0.820	− 0.467				
**Perceived trust**									
PTR1	4.20	2.761	0.881	− 0.876	− 0.218	0.937	0.941	0.955	0.842
PTR2	4.27	4.913	0.937	− 0.981	− 0.035				
PTR3	4.28	4.650	0.932	− 0.931	− 0.223				
PTR4	4.06	3.542	0.918	− 0.940	− 0.164				
**Intention to use**									
IU1	3.98	3.380	0.960	− 1.208	− 0.045	0.913	0.913	0.958	0.920
IU2	3.93	3.380	0.958	− 1.181	0.008				
**Intention to recommend**									
RECOM1	4.36	2.735	0.899	− 0.821	− 0.350	0.898	0.899	0.936	0.831
RECOM2	4.25	3.509	0.933	− 0.902	− 0.280				
RECOM3	3.95	2.535	0.902	− 0.950	− 0.074				

Harman’s single factor test was used to examine the effect of common method bias (CMB). Should a single factor have total variance above 50%, it is likely that CMB will influence the data and, consequently, the empirical outcomes (Podsakoff et al. 2003). In our study the total variance for a single factor is 47.32%. When the complete set of factors was present in the model, 76.03% of the variance was explained. This suggests that it is unlikely that common method bias exists (Molinillo et al. 2020).

### Data analysis

To evaluate the research model and the proposed hypotheses, structural equation modelling using partial least squares (PLS) was used with the SmartPLS3 software. PLS simultaneously estimates the measurement and structural parameters, although the analysis must be carried out in two stages: the analysis of the measurement model and the analysis of the structural model.

## Results

### Evaluation of the measurement model

The evaluation of the measurement model analysed its reliability and convergent and discriminant validity. Internal consistency of the measurement items was tested via Cronbach’s alpha (CA) index (Cronbach 1951) and the Rho coefficient (Nunnally and Bernstein 1994). The values of both tests were higher than the minimum recommended value of 0.7. Also, all values for composite reliability (CR) were above 0.90. Convergent validity was verified by examining the item loadings for each factor (standardised loadings) to ensure all variables were above 0.70 and significant (p < 0.00). All values for the average variance extracted (AVE) were higher than 0.50 (Hair et al. 2017). Table 3 lists these values for each variable, along with the mean of each item.

The discriminant validity of the data was then analysed to examine the different dimensions measured by each construction. Two methods were applied: first, the Fornell-Larcker criterion that analyses whether the correlations between the different dimensions were smaller than the value of the square root of AVE (Fornell and Larcker 1981), and second, the heterotrait-monotrait (HTMT) ratio that measures whether the correlations between pairs of constructions are under 0.9 (Henseler et al. 2014). Table 4 shows the results that had values close to those recommended in the scientific literature. Consequently, the discriminant validity in the model was considered satisfactory. It was also essential to analyse the possibility of multicollinearity between predictive variables. Variables that are highly correlated provide low independent explanatory capacity. Hence, the collinearity assessment test for the variance inflation factor (VIF) should present values below 5; in this study, all VIF values were below 5, and the mean VIF value was 3.181, which rules out the possibility of collinearity (Hair et al. 2017). These values are presented in Table 5.

**Table 4:** General model resolution by SmartPLS using PLS algorithm and bootstrapping

Nº	Research hypotheses	Path Coefficient	Std Dev	t-value	p-value	f^2^	Result
H1( +)	CON → IU	0.112	0.056	1.992	0.047	0.014	Supported
H2( +)	EE → IU	− 0.056	0.042	1.337	0.182	0.006	Not supported
H3( +)	SN → PU	0.576	0.043	13.421	0.000	0.495	Supported
H4( +)	SN → TR	0.505	0.053	9.564	0.000	0.342	Supported
H5( +)	PPIT → IU	0.138	0.063	2.202	0.028	0.025	Supported
H6( +)	HAB → IU	0.299	0.060	4.949	0.000	0.116	Supported
H7( +)	PU → IU	0.145	0.048	3.001	0.003	0.037	Supported
H8( +)	TR → IU	0.331	0.058	5.708	0.000	0.153	Supported
H9( +)	IU → RECOM	0.754	0.030	25.409	0.000	1.316	Supported

CON, Convenience; EE, Effort expentancy; HAB, Habit; IU, Intention to use; PTR, Perceived trust; PU, Perceived usefulness; PPIT, Personal innovation; SN, Subjective norms; RECOM, intention to recommend

**Table 5:** Discriminant validity of the measures

	CON	EE	HAB	IU	PTR	PU	PPIT	SN	RECOM
CON	**0.889**	0.714	0.768	0.775	0.789	0.706	0.863	0.725	0.518
EE	0.656	**0.966**	0.552	0.513	0.501	0.455	0.662	0.712	0.334
HAB	0.696	0.501	**0.913**	0.846	0.747	0.742	0.851	0.766	0.611
IU	0.711	0.473	0.774	**0.959**	0.834	0.751	0.832	0.738	0.581
PTR	0.730	0.466	0.696	0.775	**0.917**	0.680	0.802	0.686	0.549
PU	0.640	0.415	0.674	0.683	0.628	**0.866**	0.760	0.628	0.644
PPIT	0.780	0.603	0.768	0.754	0.737	0.686	**0.911**	0.798	0.538
SN	0.672	0.665	0.706	0.685	0.646	0.579	0.734	**0.919**	0.459
RECOM	0.470	0.305	0.549	0.524	0.505	0.576	0.482	0.421	**0.869**

Note. Main diagonal: in bold square root of the AVE

Fornell-Larcker criterion (below the main diagonal) and HTMT ratio (above the main diagonal)

CON, Convenience; EE, Effort expentancy; HAB, Habit; IU, Intention to use; PTR, Perceived trust; PU, Perceived usefulness; PPIT, Personal innovation; SN, Subjective norms; RECOM, intention to recommend

### Evaluation of the structural model

The analysis of the structural model was performed (without considering the moderating effect of Covid-19; see Table 4). First, the research hypotheses were tested through comparative analysis of the structural coefficients. In addition, the coefficients of determination R^2^ (explained variance) and f^2^ (effect size) that predict capabilities and relationships between the constructs (Hair et al. 2017) were assessed. According to Hair et al. (2017) and Henseler et al. (2016), f^2^ values above 0.35, 0.15 and 0.02 can be considered strong, moderate and weak, respectively. Based on this metric, H1 (β CON → IU = 0.112, p-value = 0.014), H5 (β PPIT → PU = 0.138, p-value = 0.028) and H7 (β PU → IU = 0.145, p-value = 0.037), were supported but the f2 effect size suggested only a low effect of convenience, personal innovation and perceived usefulness on intention to use. This finding indicates that these variables had a significant relation with intention to use, but a small impact on it. The rest of the variables had a moderate (H6, H8) or strong (H3, H4, H9) effect. The results confirmed that habit (β HAB → IU = 0.299, p-value = 0.000) and perceived trust (β PTR → IU = 0.331, p-value = 0.000) have an impact on intention to use. Also, subjective norms are significant on perceived usefulness (β SN → PU = 0.576, p-value = 0.000) and perceived trust (β SN → PTR = 0.505, p-value = 0.000). In addition, intention to use H9 (β IU → RECOM = 0.754, p-value = 0.000) confirmed a positive impact on intention to recommend. Lastly, H2 (β EE → IU = −0.056, p-value = 0.182) is not significant, so effort expectancy appears not to have an impact on intention to use.

The latent variable, R^2^, for intention to use was 0.735 in the general framework, which meant that convenience, personal innovation, habit, perceived usefulness and perceived trust explained 73.5% of intention to use. It also important to check Stone-Geisser’s predictive relevance (Q^2^). This can be achieved by making use of the blindfolding procedure in SmartPLS. According to Chin (1998), a model demonstrates good predictive relevance when its Q^2^ value is larger than zero. Also, the value of the standardized mean squared residual (SRMR) ratio was analysed to contrast the difference between the observed correlation and the predicted correlation as an indicator of model fit (Henseler et al. 2014). The value is 0.045, which is below the recommended threshold of 0.08. Finally, the NFI is 0.854, which is very close to the optimum value. Table 6 summarizes all of the values.

**Table 6:** R^2^ and Q^2^ for all Dependent Variables and model fit indices

	Intention to use	Perceived trust	Perceived usefulness	Intention to recommend
R^2^	0.735	0.255	0.331	0.568
Q^2^	0.662	0.210	0.243	0.467
SRMR	0.045			
NFI	0.854			

### Moderating effect of COVID-19

First, the measurement invariance assessment procedure (MICOM) was applied (Henseler et al. 2016) to determine “whether, under different conditions of observation and study of phenomena, measurement models yield measurements of the same attribute” (Henseler et al. 2015). It is a three-step procedure to evaluate the measurement invariance of variables: configural invariance, compositional invariance and composite equality. Configural invariance is a precondition for compositional invariance, which is also a precondition for a meaningful assessment of composite mean.

Byrne et al. (1989) proposed the concept of partial measurement invariance. Partial measurement invariance applies to factors that are configurationally invariant, and the problem first arises when measurement invariance is imposed on the model. According to Henseler et al. (2016) if a configural (step 1) and compositional (step 2) invariance is set, we can speak of a partial measurement invariance; otherwise, no measurement invariance is established. In this case, the partial invariance is confirmed because steps one and two conform to the limits established in the literature while step three does not find optimal values in all variables. However, in practical applications, the complete measurement invariance is often not met (Steenkamp and Baugarner 1998).

After the measurement invariance was checked, the structural model was estimated, using the PLS multigroup analysis method (Henseler et al. 2009). The main idea of this analysis is to check whether the path coefficients differ significantly across the two groups. Table 7 shows the results of the differences between groups test for all of the relationships within the model. As shown in Table 7, two relationships (H3 and H4) differ significantly across the two groups. All other path coefficients do not differ significantly. Therefore, the results indicate that fear of COVID-19 moderates the relationship between subjective norms regarding perceived usefulness and perceived trust.

**Table 7:** Moderating Effect of COVID-19

Nº	Research hypotheses	Path coefficient (with fear)	Path coefficient (without fear)	Coefficients-diff	p-value
H1( +)	CON → IU	0.149	− 0.027	0.176	0.130
H2( +)	EE → IU	− 0.089	0.028	− 0.117	0.165
H3( +)	SN → PU	0.684	0.329	0.355	**0.000**
H4( +)	SN → PTR	0.614	0.211	0.357	**0.001**
H5( +)	PPIT → IU	0.125	0.173	− 0.049	0.706
H6( +)	HAB → IU	0.292	0.324	− 0.032	0.815
H7( +)	PU → IU	0.122	0.211	− 0.089	0.430
H8( +)	PTR → IU	0.344	0.316	0.028	0.817
H9( +)	IU → RECOM	0.728	0.807	− 0.080	0.210

Note. Significant differences are shown in bold

CON, Convenience; EE, Effort expentancy; HAB, Habit; IU, Intention to use; PTR, Perceived trust; PU, Perceived usefulness; PPIT, Personal innovation; SN, Subjective norms; RECOM, intention to recommend

## Discussion

### Theoretical conclusions

Our findings provide some useful theoretical contributions. The study proposed was novel because there are no known studies analysing the intention to recommend biometric mobile payment systems such as the one considered in the present pandemic situation, where in addition to analysing its background, the analysis of the moderating effect of fear towards the COVID-19 has been included. Most studies in the literature address pre-adoption behaviour by users and the intention to use classical payment systems (Kaur et al. 2020; Liébana-Cabanillas et al. 2020b; Lin et al. 2020). However, this research focused on the study of the intention to recommend a highly innovative and PSD2-compliant system (Polasik et al. 2020). The results of the present research demonstrate the influence of a set of variables on the intention to recommend based on the S-O-R model. Based on this approach, the antecedents that determine the intention of use were, first, perceived trust; second, the user’s habit of handling technologies; third, personal innovation; and finally, convenience of use. Moreover, the intention to use is a key determinant of the intention to recommend.

Specifically, convenience has a positive influence on the intention to use (Maziriri et al. 2020), as users consider this new payment system more efficient than conventional payment systems such as cash or other more current ones such as NFC or QR payment systems. On the other hand, it is also verified that subjective norms have a positive relationship on perceived usefulness and perceived trust, in line with the proposals of Lara-Rubio et al. (2020) and Yasin et al. (2019), who found that the influence of the opinions of people close to the users improve the perceived usefulness of the proposed payment system, as well as the perceived trust—that is, that the opinions of people with closer contact with the users will improve usefulness and trust.

In parallel, the importance of the antecedents of intention to use, personal innovation, habit, usefulness and perceived trust is also corroborated. With respect to the personal innovation of the users, a greater capacity for innovation among users will improve the intention to use the proposed payment system (Patil et al. 2020). On the other hand, the habit that the users manifest with the repetitive use of new technologies such as the one proposed improves intention of use (Jia et al. 2020). The manifest utility of payment systems with ocular iris scanning, where payments are made in a simpler and faster way than with other payment tools, increases intention to use, in line with previous research (Liébana-Cabanillas et al. 2020a). Finally, improved trust additionally influences consumer intention to adopt these new payment systems (Kalinic et al. 2019b).

Finally, the recommendations will improve from the higher usage intention among consumers and users of payment systems with ocular iris scanning (Talukder et al. 2019).

To conclude this analysis of results, the importance of COVID-19 among the users surveyed was contrasted. We are facing a completely new situation that puts one’s health at risk. The moderating effect of COVID-19 shows that the most fearful users will be more influenced by the comments of the people around them than those who are not afraid. In this respect, they value more intensely the opinion of their social group regarding the usefulness of this payment system and the trust it can generate. It has thus been demonstrated that the proposed model makes it possible to analyse the intention to recommend this biometric mobile payment system and how the fear of contagion from the use of other payment systems determines some of the proposed relationships.

### Implications for management

The use of smartphones has increased considerably in recent years due to the improvements in the functionality they offer their users. One of the implemented functionalities is the payment/collection of purchases made in the new economic and social environments where users carry out their economic activity. Mobile phone payment is one of the major trends in the business sector, which is why the companies in charge of developing hardware and software allocate significant funds to promote their use. It is precisely within this new context—in which payment regulations are changing to offer greater security to users (PSD2) and the effect of the COVID-19 pandemic on people’s lives—that the use of mobile phones has aroused growing interest. Payment with alternative systems to traditional cash such as bank cards and mobile phones is thus expanding the customer base. At the same time, biometric payment options (e.g., hand geometry or fingerprints) ensure greater financial security for users, but do not guarantee infection-free payment. Considering this situation, a mobile phone–based payment option that carries out the verification of the user through scanning his or her iris guarantees both financial and physical security, which is so much in demand in during this time of crisis.

This study found that an iris recognition scanning mobile payment system would be highly recommended among users based on the intention to use that it arouses. This intention to use is very clearly influenced by perceived trust, habit, personal innovation and convenience of use. Regarding perceived trust, companies providing this type of payment system must design communication campaigns aimed at promoting the guarantees that this system has for its users, where payment security is absolute as the user is uniquely identified and there is no possibility of fraud or impersonation in the event of the theft of a card or mobile phone. In this context, opinion dynamics is a useful tool to model the diffusion and opinion evolution (Zha et al. 2020). At the same time, given that most users are used to using new technologies, it will also be essential to convey that this payment system is a new technical tool that will guarantee greater peace of mind when making a payment in an establishment, so that the personal habit for innovation will reinforce the intention to use the system and subsequently recommend it. Finally, it will also be essential to provide information on the suitability of using this type of mobile payment system to guarantee confidence and increase the intention to use.

Marketing companies and payment service providers should also take into account the importance of subjective norms. Today, consumers are highly connected and value recommendations made to adopt a technology in a very positive and quasi-taxing way. Feedback from society, families and closed groups is really significant and influential for users. Companies can shape these opinions by focusing on the effective promotion of a biometrics charging service. Service providers offering these payment systems should thus foster a positive environment with respect to this technology by promoting their main features and benefits, which will drive the intention to use.

The influence of fear of contagion in the current pandemic situation was analysed, and it was found that users who have a greater perception of risk would perceive to a greater extent the relationship between subjective norms and trust and subjective norms and utility. This reinforces previous studies that highlight how subjective norms and trust largely determine the adoption of a new technology, which in the present case is conditioned by an extreme health situation. It will be essential to provide suitable information on the advantages of the proposed payment system compared with other payment systems where there could be physical contact between the buyer and the seller. Especially within the context of the COVID-19 pandemic, it is recommended that interested organisations encourage users to share their experience with biometric payment methods with their friends and social group. If the objective is to extend the use of these methods, potential users who are most afraid of contagion will consider their social group’s opinion and good references regarding such systems’ usefulness and trustworthiness to be essential. Encouraging current users to share their experience with this payment method in their own social networks will help to expand the service more quickly as it will be seen by their social group. Likewise, encouraging users to share their opinions on the organisation’s website, in forums or blogs will also help in this process.

### Limitations and future lines of research

One of the main limitations of the study is that the sample is made up of potential Spanish users of this technology. Future research could include users from other countries to generalise the theoretical conclusions reached in this study. Another limitation is the type of online survey based on a structured questionnaire to determine the recommendation intention of the surveyed users at a given time. Future research could use longitudinal data and a mixed-method approach with qualitative techniques such as consumption narratives (Rahmanian 2021) and more than one survey technique (Guinalíu and Díaz de Rada 2020). This research was carried out in Spain, which has one of the highest infections and mortality rates in the world, and this may have conditioned respondents’ responses. It would be advisable to analyse samples from countries with lower infection and mortality rates to see if the results are consistent.

Finally, the authors of this study also suggest that future studies be conducted to analyse the problems in iris recognition and moderating effect of other socio-demographic variables that would allow for the segmentation of potential users to ensure a higher recommendation rate.

## Data Availability

Not applicable.

## Appendix: Scales

**Convenience** (Pal et al. 2015; Liébana-Cabanillas et al. 2019)

The displayed payment system is convenient (CON1)

The displayed payment system is convenient because I can use it at any time (CON2)

The displayed payment system is convenient because I can use it in any situation (CON3)

The displayed payment system is convenient because it is not complex (CON4)

**Effort expectancy** (Venkatesh et al. 2012; Hew et al. 2015)

Learning to use the displayed payment system is easy for me (EE1)

I find the displayed payment system easy to use (EE2)

**Subjective norms** (Taylor and Todd 1995; Kalinić et al. 2019)

People whose opinions I value would approve of me using the displayed payment system to buy products (SN1)

I consider that most people should use the displayed payment system to buy products (SN2)

I am expected to use the displayed payment system to buy products (SN3)

People close to me would agree to my using the displayed payment system to buy products (SN4)

**Personal innovation** (Ramos de Luna et al. 2016; Kalinić et al. 2019)

I like to test new technologies (PIIT1)

Among my friends and family, I am usually the first to try out new information technologies (PIIT2)

In general, I would not hesitate to test new technologies (PIIT3)

I would like to look for new ways to test new technologies (PIIT4)

**Habit** (Cheng et al. 2020)

The use of new technologies has become a habit for me (HAB1)

I am addicted to using new technologies (HAB2)

I must use new technologies (HAB3)

**Perceived usefulness** (Ramos de Luna et al. 2016)

Using the displayed payment system could help me to make the purchases I normally make on the Internet (PU1)

Using the displayed payment system could increase my efficiency when making purchases (PU2)

Using the displayed payment system for my purchases could increase my productivity (PU3)

In general, the displayed payment system could be useful for me when making my purchases (PU4)

**Perceived trust** (Zarmpou et al. 2012)

The payment system displayed is reliable (TR1)

I would rate the displayed payment system as honest (TR2)

I think the payment system displayed is responsible (TR3)

In general, I trust the payment system displayed (TR4)

**Intention to use** (Liébana-Cabanillas et al. 2020b)

Accepting that I have access to the payment system shown, I intend to use it to make my purchases (IU1)

If I had access to the payment system shown over the next few months, I believe that I would use it, rather than an alternative system (IU2)

**Intention to recommend** (Ma et al. 2017)

I would like to recommend the displayed payment system to people (RECOM1)

I have many reasons for recommending the display payment system (RECOM2)

I would recommend the displayed payment system to people through different channels (RECOM3).

## Fear

Are you afraid of getting infected through using traditional payment systems such as cash or bank cards?

## Footnotes

1 https://ec.europa.eu/info/law/payment-services-psd-2-directive-eu-2015-2366_es.

2 A joint venture of Caixa Bank, Global Payment, Visa, Samsung and Arval that specialises in payment gateways and business solutions.
